# GWAS reveals common *SLX4* variants associated with telomere length and hypertension in individuals of African ancestry

**DOI:** 10.1007/s13258-026-01746-y

**Published:** 2026-02-20

**Authors:** Nicole Benfield, Prisca K. Thami, James Ware, Laura Collopy

**Affiliations:** National Heart and Lung Institute, Imperial College London, Guy Scadding Building, Cale Street, South Kensington, London, SW3 6LY UK

**Keywords:** Human genomics, Telomere length, Hypertension, GWAS, Ethnic disparities

## Abstract

**Background:**

Cardiovascular disease (CVD) and its risk factors are highly prevalent in the UK, with incidence differing by ethnicity. Black individuals have an increased prevalence of heart failure, stroke and hypertension compared to White individuals. Telomere length has been associated with many age-related conditions, with shorter telomeres associated with increased CVD risk. However, Black individuals have longer telomeres on average compared to White individuals, which should be protective against CVDs. The increased incidence of CVD in this population indicates a paradoxical association.

**Objectives:**

To investigate the relationship between telomere length and cardiovascular disease risk across ethnic groups and to identify genetic variants that may explain observed differences.

**Methods:**

Using the UK Biobank cohort, we assessed telomere length in Black and White populations and performed GWAS analysis to identify ethnicity-specific variants associated with telomere length.

**Results:**

We report that Black individuals had significantly longer telomeres than White individuals (0.9035 vs 0.8301, *p* < 0.0001). Contrary to published reports, Black individuals showed lower incidence of most CVD subtypes compared to White individuals. However, hypertension was more prevalent in Black individuals despite longer telomeres. GWAS analysis identified 12 ethnicity-specific variants at genome-wide significance associated with longer telomeres in Black populations. Notably, 3 variants in *SLX4* were significantly more common in Black individuals and associated with both longer telomere length and hypertension risk. High BMI and lower household income were found to influence hypertension risk, overriding the protective effect of longer telomeres in Black populations.

**Conclusions:**

The increased rate of hypertension in Black populations, as well as longer telomere length, may be influenced by common genetic variants. The protective effect of longer telomeres against CVD does not extend to hypertension, where socioeconomic and metabolic factors appear to override this protection**.**

**Supplementary Information:**

The online version contains supplementary material available at 10.1007/s13258-026-01746-y.

## Introduction

Cardiovascular disease (CVD) describes many different heart and blood vessel related pathologies, such as ischemic heart disease, cerebrovascular disease, peripheral artery disease, and aortic atherosclerosis (Lopez et al. 2022). CVD is a leading cause of mortality worldwide (https://www.who.int/publications/i/item/9789240074323), with Public Health England reporting 1 death every 4 min in England, costing the economy £15.8 billion annually, with an estimated £7.4 billion of this in healthcare costs (https://www.gov.uk/government/publications/health-matters-preventingcardiovascular-disease/health-matters-preventing-cardiovascular-disease#). Many factors increase CVD risk including excessive alcohol consumption, smoking, poor diet, obesity, dyslipidaemia, type 2 diabetes (T2D), and hypertension (Stewart et al. 2017). Age and gender are also independent risk factors for CVD, with risk increasing with age, and post-menopausal females being at greater risk than age-matched men (Rodgers et al. 2019), due to increased oxidative stress and production of reactive oxygen species, increased inflammatory signal molecules production (Curtis et al. 2018), as well as the decline of sex hormones oestrogen and testosterone (Villa et al. 2015; Ruige et al. 2011).

Critically, CVD prevalence and risk differs substantially by ethnicity (Schultz et al. 2018; Scarborough et al. 2010; Tillin et al. 2013), with CVD risk higher among South Asian and Black populations compared to White populations in both UK Biobank (UKB) cohort studies (Ho et al. 2022) and US populations (Mazimba and Peterson 2021). Environmental factors and socioeconomic inequalities (measured by income, education level and employment status) are strongly associated with increased CVD risk (Schultz et al. 2018; Tillin et al. 2013) and are disproportionately prevalent in minority populations. Low income is thought to increase CVD risk through reduced access to high-quality healthcare (Stirbu et al. 2012), with individuals of lower income less likely to have regular health check-ups, leading to poorly managed CVD risk factors including hypertension and T2D (Parikh et al. 2014). Understanding how genetic and environmental factors interact to produce these health disparities is essential for developing targeted interventions.

Telomere length (TL) has been associated with many age-related health conditions (Bountziouka et al. 2022), with shorter telomeres correlating with increased CVD risk (Yeh and Wang 2016; Gebreab et al. 2017). TL is a complex trait influenced by genetic, environmental (Zglinicki 2002) and social factors (Codd et al. 2021). Recent large-scale genomic studies have substantially advanced our understanding of TL genetics. Codd et al*.* (2021) identified 197 independent genetic variants at 138 loci associated with TL in 472,174 UKB participants using qPCR measurements (Codd et al. 2021), while Burren et al. (2024) combined qPCR and whole-genome sequencing data from 462,666 participants to identify 64 rare variants and demonstrate that rare variants have larger effects on TL than common variants (Burren et al. 2024). These studies identified genes involved in telomere regulation and maintenance, including components of the shelterin and CST complexes, and telomerase formation and activity.

However, these predominantly European-ancestry studies have not addressed a critical paradox relevant to health disparities: average TL is longer in Black individuals compared to White individuals (Hunt et al. 2008; Lynch et al. 2016; Raymond et al. 2016; Hansen et al. 2016), yet CVD incidence is reported to be greater in Black populations (Benetos and Aviv 2017; Selvaraju et al. 2021). This apparent contradiction (longer telomeres that should be protective, yet higher disease burden) has remained unexplained. Furthermore, whether ethnicity-specific genetic variants contribute to TL differences between populations, and how genetic predisposition interacts with environmental and socioeconomic factors to determine disease outcomes, remains poorly understood.

In this study, we leverage UK Biobank whole-exome sequencing data to investigate TL and CVD across Black and White populations, with three specific aims: to identify ethnicity-specific genetic variants associated with longer TL in Black individuals; to test whether the reported telomere paradox holds true across different CVD subtypes; and to determine whether environmental and socioeconomic factors modify the protective effects of longer telomeres. We report that longer TL is indeed protective against most CVD subtypes in Black populations, resolving the apparent paradox. However, we identify hypertension as a notable exception, where variants in *SLX4* predispose to both longer TL and increased hypertension risk, and where environmental factors (lower income, higher BMI) override telomere-mediated protection. Our findings demonstrate the critical importance of considering gene-environment interactions and population-specific genetic architecture in understanding and addressing cardiovascular health disparities.

## Results

### TL is significantly different between ethnicities in the UKB cohort

**Fig. 1:**
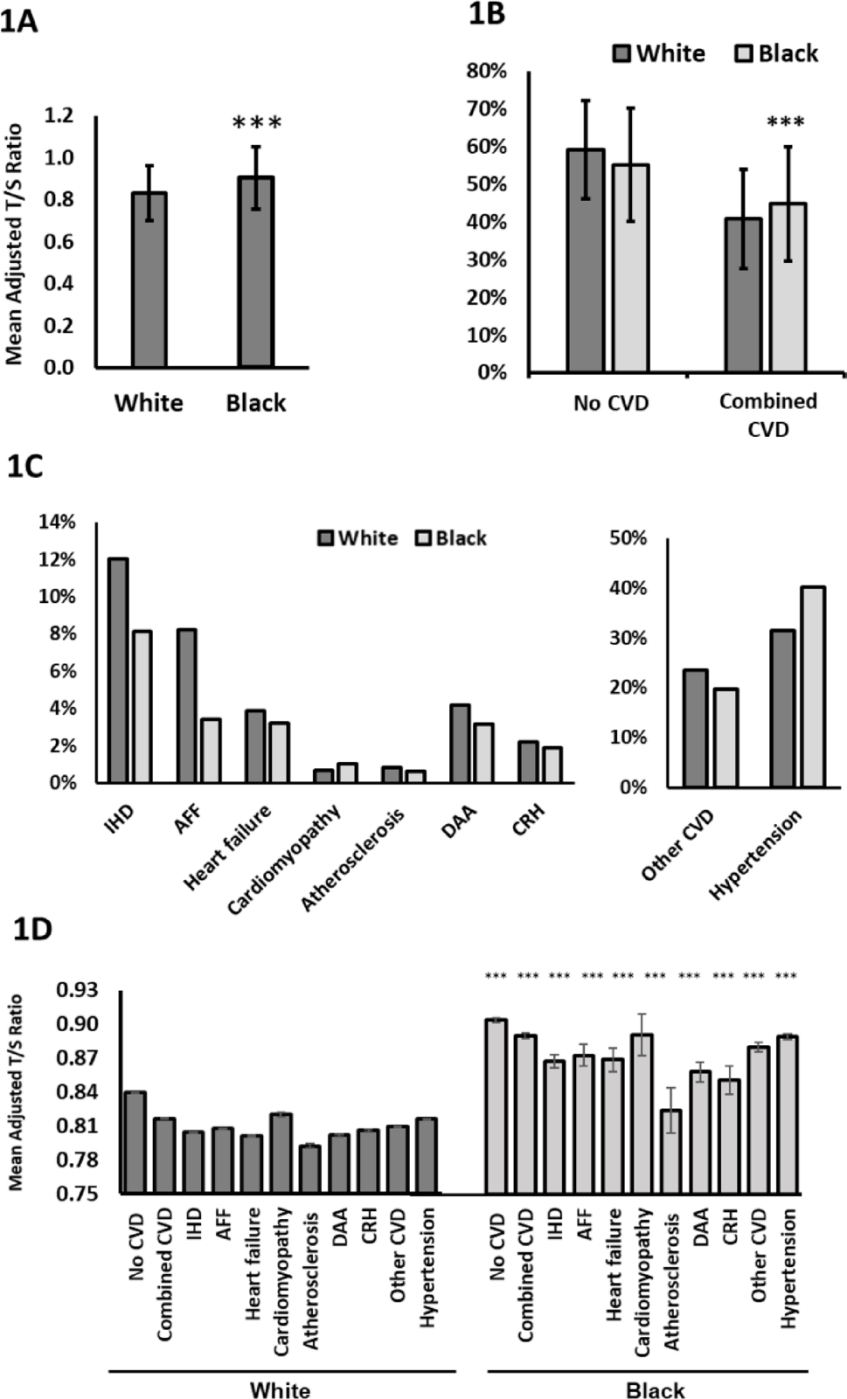
Longer telomere length in Black individuals is protective against CVD. **A** Mean adjusted T/S ratio per cohort, as determined by Codd et al*.* (2022). White n = 445,408; Black n = 7280. **B** Incidence of combined CVD in Black and White participants for whom telomere length data was available. Shown as percentage of n = 445,408 (White) and n = 7280 (Black). **C** Incidence of CVD subtypes in Black and White participants for whom telomere length data was available. Shown as percentage of n = 445,408 (White) and n = 7280 (Black). **D** Mean adjusted T/S ratio in Black and White individuals presenting with CVD. All error bars are SEM. *** = *p* < 0.001, unpaired t-test

Filtering individuals in the UKB cohort to those including ‘Adjusted T/S Ratio | Instance 0’ measurements and by ethnicity, resulted in 445,408 White individuals and 7280 Black individuals for this study. To investigate the differences in TL between ethnicities, mean adjusted T/S ratio values were compared between the two cohorts. TL were significantly longer in Black individuals (0.9035 ± 0.1497) compared to White individuals (0.8301 ± 0.1302, *p* < 0.0001, Fig. 1a). This data echoes previous findings. Hansen et al*.* (Hansen et al. 2016) proposed White individuals have shorter TL on average as shorter telomeres are protective against melanoma. This is supported in our data, which shows that White individuals with a melanoma diagnosis have significantly longer telomeres (Figure S1).

### Longer telomeres protect against many types of CVD

Previous studies have identified differences in CVD prevalence and risk between ethnicities (Scarborough et al. 2010; Tillin et al. 2013; Ho et al. 2022; Mazimba and Peterson 2021). To investigate this in the UKB cohort, summary data for hospital inpatient records were used to identify individuals with reported CVD. A greater percentage of Black individuals have combined CVD (Fig. 1b). However, this is skewed by the high incidence of hypertension in this population. Breaking CVD into sub-types, incidence of ischaemic heart disease, atrial fibrillation and flutter, heart failure, atherosclerosis, diseases of arteries, arterioles and capillaries and chronic rheumatic heart disease, are all lower in Black individuals versus White (Fig. 1c). Black and White individuals with these conditions also have shorter telomeres, on average, than those with no CVD diagnosis (Fig. 1d). This indicates that, contrary to published data, Black people have a lower incidence of CVD compared to White, except for primary hypertension, and longer TL is indeed protective.

### Variants in SLX4 predispose black populations to both longer TL and hypertension

To investigate whether Black populations have a genetic predisposition to longer telomeres, GWAS were carried out using WES data. The Black GWAS was underpowered due to the limited Black population included within the UK Biobank (n = 5849). This demonstrates the need for improved ethnic diversity in biobanks (Supplementary Fig. 2a). The White population GWAS (n = 290,366) identified 106 top loci, or sentinel variants (Supplementary Table S1, Figure S2B). Eighteen of the variants had been previously identified and 87 were novel. Of the novel variants, 38 are in known loci associated with telomere maintenance and regulation. For example, *ACD, TERF1* and *POT1* encode proteins involved in the shelterin complex (Xin et al. 2008); *CTC1* and *STN1* encode proteins in the CST complex (Makarova and Kulbachinskiy 2012); *RTEL1* is required for telomere length regulation (Uringa et al. 2011); while *TERT*, *NAF1* and *SHQ1* are involved in telomerase assembly and activity (Grozdanov et al. 2009; Venteicher et al. 2008). Genes involved in DNA replication and DNA damage repair were also identified, including *POLI* (Makarova and Kulbachinskiy 2012) and *RPA1* (Haring et al. 2008*).* The *HBB* loci identified is a known artefact as the *HBB* gene is used to quantify leucocyte TL T/S ratio by Codd et al*.* (2022), thus this association with TL is not considered further (Codd et al. 2022).

**Fig. 2:**
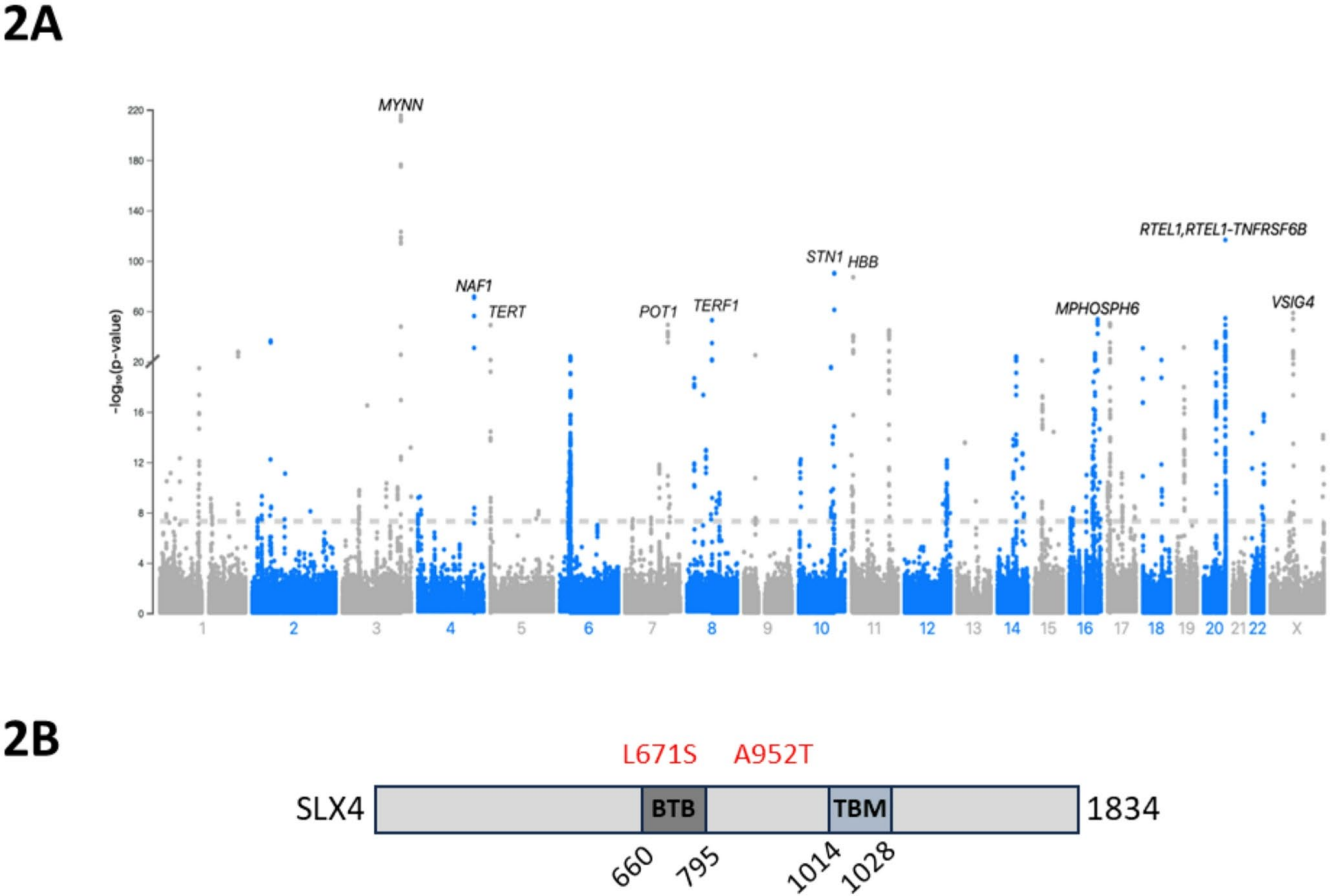
Variants at 12 loci are associated with longer TL in Black individuals. **A** Manhattan plot highlighting the regions containing 12 sentinel variants of genome-wide significant in the Black UK Biobank population (n = 296,215, Horizontal dashed reference line = GWAS significance threshold). See supplementary Figure S2C for QQ plot. **B** Schematic showing domain mapping of human SLX4 protein, indicating the position of two coding variants significantly associated with longer TL in Black individuals, and hypertension Gaciong et al,. (2013). BTB, Bric-a-brac, tramtrack and broad complex domain; TBM, TRF2-binding motif

As the GWAS in the White population replicated loci associated with TL in the literature, we combined the Black and White populations in one dual-ethnicity GWAS and compared the results with the White-only analysis, with the aim of identifying loci associated with TL that may have Black-specific variants. Of the 112 top loci with genome-wide significance (*p* < 5 × 10–8), variants in 12 different genes differed between the two GWAS (Fig. 2 and Table 1). We investigated the variant allele frequency for each of these variants in the Black and White populations and confirmed their significance Fisher’s exact test. All were confirmed to be significantly more common in Black individuals versus white (*p* < 0.0001, Fisher’s exact test, Table 1). These variants may therefore have particular significance for telomere length in the Black population.

**Table 1:** Top significant loci associated with TL from dual-ethnicity GWAS that were not present in white-only GWAS

Marker	rsID	Nearest gene	Variant type	*P* value	White VAF	Black VAF
17: 7,848,460 G/A	rs3744249	*KDM6B*	Synonymous	7.10E-51	0.1095	0.2964
16: 74,660,833 C/A	rs79470274	*RFWD3*	Intronic	3.86E-27	0.1381	0.0586
19: 22,286,431 G/C	rs808373	*ZNF729*	5’ UTR	1.12E-17	0.4961	0.4620
17: 8,161,461 C/G	rs75664430	*VAMP2*	3’ UTR	1.38E-17	0.2531	0.1501
11: 5,508,635 G/A	rs186434	*AC104389.5, UBQLN3*	Synonymous	1.85E-16	0.9996	0.9448
11: 4,923,560 C/T	rs59534473	*MMP26*	Intronic	1.08E-11	0.0001	0.0958
3: 128,487,017 G/C	rs1573858	*GATA2*	Synonymous	4.84E-11	0.6196	0.8030
17: 44,848,191 C/T	rs12164	*HIGD1B*	Synonymous	3.56E-09	0.0698	0.1142
2: 168,834,219 T/C	rs522893	*NOSTRIN*	Splice	8.09E-09	0.0560	0.0807
14: 95,713,905 C/A	rs1122138	*TCL1A*	Intron	1.32E-08	0.1559	0.3680
16: 3,595,606 A/G 16: 3,590,784 C/T 16: 3,600,969 G/A	rs77985244 rs59939128 rs80116508	*SLX4*	Missense Missense Intronic	2.93E-08 3.64 × 10–8 3.88 × 10–8	0.0642 0.0640 0.0642	0.0875 0.1065 0.0724
16: 90,074,947 A/C	rs12925933	*PRDM7*	Missense	3.29E-08	0.6768	0.1679

All variants were identified from GWAS analysis with a *P*-value < 5 × 10–8. VAF; variant allele frequency

From this list, *SLX4* is particularly interesting. Three variants were identified at genome-wide significance (*p* < 5 × 10^–8^) (Table 1, Fig. 2a and b) and these have a significantly higher frequency in the Black population. *SLX4* is a tumour suppressor that acts as a scaffold for many proteins, playing a pivotal role in DNA repair and maintenance of genome stability. It also has a known role in telomere maintenance (Sarkar et al. 2015), and loss-of-function variants are associated with the premature ageing syndromes Fanconi anaemia and Hoyeraal-Hreidarsson (Kim et al. 2011). The variant rs77985244 (p.Leu671Ser), identified in this GWAS, lies in the BTB domain, which associates with RTEL1*.* This interaction is required for optimal DNA replication and drives recruitment of RNA pol II during transcription (Uringa et al. 2011). Further, *SLX4* must homo-dimerise via its BTB domain to function and localise to telomeres, and as the substituted serine can hydrogen bond (a stronger interaction than non-polar interactions) (Yin et al. 2016), dimerization could be encouraged leading to enhanced telomeric localisation and contribute to more stable or longer telomeres in the Black population.

A second missense variant in *SLX4* was also identified to be significantly more prevalent in the Black population (rs59939128, p.Ala952Thr), and this lies between the BTB domain and TRF-2 binding region of SLX4 (https://www.emro.who.int/media/world-health-day/public-health-problem-factsheet-2013.html)*.* The third *SLX4* variant identified, rs80116508 (c.1163 + 10C > T), is intronic (previously reported by Codd et al., 2021). Remarkably, rs77985244 and rs59939128 have also been associated with hypertension in a previous GWAS, in addition to their association with longer TL in Blacks, identified here (He et al. 2019). Data therefore suggests that key variants in *SLX4* may predispose to both longer TL and hypertension, contributing to the incidence of both of these phenotypes in the Black population.

### Environmental factors linked to hypertension are more prevalent in the black population

Given that longer TL is protective against multiple types of CVD in the Black population, but hypertension is seen more frequently in Black than White, we hypothesised that, in addition to the key variants in *SLX4,* Black individuals have an increased risk of hypertension due to environmental factors which counteract the potential protective impact of longer telomeres. To investigate this, we looked at household income and BMI as risk factors known to be associated with hypertension (Leng et al. 2015; Landi et al. 2018).

**Fig. 3:**
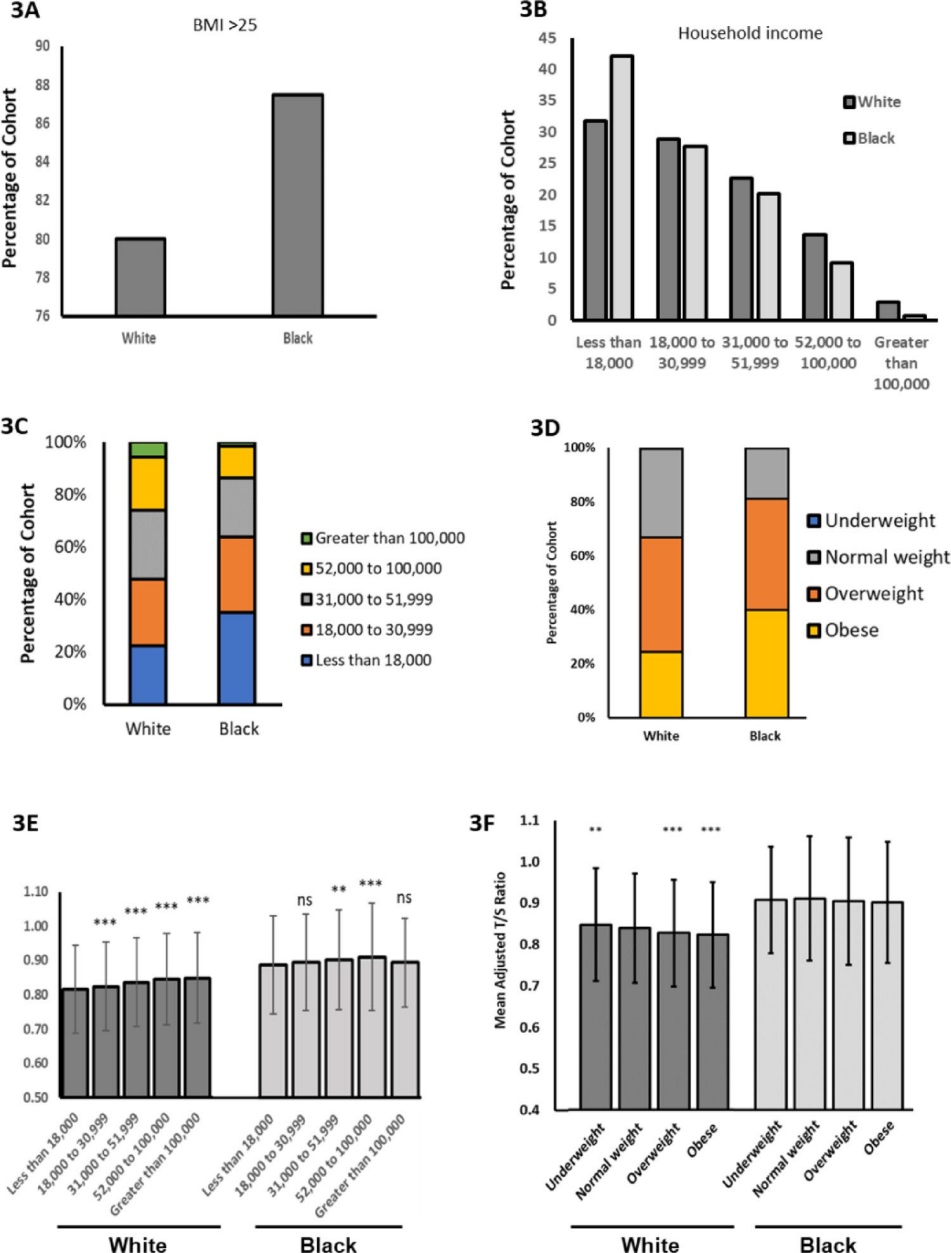
Environmental factors more common in the Black population increase hypertension and decrease telomere length. **A** Percentage of each cohort with hypertension and a BMI > 25. Total hypertensive cohort White n = 119,016; Black n = 2295. **B** Percentage of each cohort with hypertension under each income bracket. **C** Percentage of total cohort within each income bracket. White n = 445,408; Black n = 7280 **D** Percentage of each total cohort overweight and obese. **E** Mean adjusted telomere length for each cohort per income bracket. **F** Mean adjusted telomere length for each cohort per BMI

Lower income, fewer educational qualifications and lack of employment are all associated with hypertension (Leng et al. 2015), as is a BMI over 25 (Landi et al. 2018). Within the UK Biobank, for both ethnicities the percentage of individuals with hypertension is greater among those who have a BMI over 25 (considered overweight or obese, Fig. 3a) and a lower household income (Fig. 3b). This supports previous findings that these factors influence hypertension risk. Other socioeconomic factors have not been presented here due to the limited number of Black individuals from the UK Biobank from which data could be obtained.

A greater proportion of the Black UK Biobank population have a low income and high BMI versus White, (Fig. 3c and d). Given that these factors are significantly increased in individuals with hypertension, and hypertension is more prevalent in the Black population, it stands to reason that these factors contribute to the increased hypertension in this population.

Mean TL is seen to increase with income (Fig. 3e) and decrease with BMI (Fig. 3f). These impacts are more pronounced in Whites, but this is likely due to the much larger population size in this study, producing results of greater significance. These trends can also be seen when the cohort is stratified by age (Supplementary Figs. 3 and 4). Nonetheless, these data demonstrate that low income and high BMI predispose to hypertension, and also have negative impact on TL. Low income and high BMI are more prevalent in the Black population, negatively impacting TL and predisposing to hypertension in this group. Crucially, the effect of these factors seems to override the protective impact of longer telomeres in the Black population.

## Discussion

We have shown in the UKB cohort of individuals with leucocyte TL measurements available that Black individuals have significantly longer TL on average than White individuals, agreeing with previous studies (Hunt et al. 2008; Lynch et al. 2016; Raymond et al. 2016). However, we also report the published paradoxical association between TL and CVD is not seen in this UKB cohort, with longer telomeres being protective against many subtypes of CVD and a lower incidence reported in the Black population. This opposes smaller studies, which have shown CVD to be more prevalent in Blacks (Nayak et al. 2020). However, incidence of hypertension remained higher in Blacks, despite their longer telomeres.

Our GWAS analysis of the White population identified many genetic loci previously associated with TL, each with key roles in telomere maintenance and regulation. Further, we also identified Black-specific variants of significance associated with longer TL. Of particular interest were variants in *SLX4,* where three variants were present at genome-wide significance (*p* < 5 × 10–8).

Hypertension is considered a global public health issue by the World Health Organisation (WHO) (https://www.emro.who.int/media/world-health-day/public-health-problem-factsheet-2013.html), causing approximately 75,000 deaths in the UK in 2015 (https://www.gov.uk/government/publications/health-matters-combating-high-bloodpressure/health-matters-combating-high-blood-pressure). It is one of the most important risk factors for CVD, in particular stroke and ischemic heart disease, associated with 51% of stroke cases worldwide (Gaciong et al. 2013). We have shown Black individuals have a higher prevalence of hypertension compared to White individuals in the UKB and this is likely to be due to both genetic and environmental predisposing factors.

Black individuals have lower incomes on average compared to White and have greater levels of obesity (Fig. 3a and b). Hypertension risk increases with low income and high BMI (Fig. 3c and d). Although these factors likely contribute to the increased incidence of hypertension in the Black population, hypertension is a complex trait and GWAS analyses have identified many associated loci. Remarkably, we identified variants in *SLX4* associated with longer TL in Blacks, that have previously been identified as predisposing to hypertension. He et al*.* (2019) predict that *SLX4* variants, including rs77985244 (p.Leu671Ser) and rs59939128 (p.Ala952Thr), modify gene expression in coronary artery, multiple brain tissues, and the right atrial appendage of the heart. In particular, they predict that high expression of *SLX4* in the cerebellum, a region implicated in the control of blood pressure through the cerebellar adrenomedullary system, may impact hypertensive phenotypes in carriers (He et al. 2019).

We further hypothesise that these variants may impact TL through the substitution of a non-polar to a polar amino acid. *SLX4* must homo-dimerise via its BTB domain to function and localise at telomeres (Yin et al. 2016), and as the substituted residues can hydrogen bond (a stronger interaction than non-polar interactions) (Alberts et al. 2002), dimerization could be encouraged leading to telomeric localisation and contribute to more stable or longer telomeres in the Black population. This requires further investigation through functional studies. As SLX4 has multiple functions, it is plausible that multiple phenotypes are affected by these variants, and they may predispose to both hypertension and long telomeres.

A meta-analysis of GWAS by Kato et al*.* (2011) identified many loci associated with hypertension risk in individuals with African ancestry, including *ARMC5, GRK4* and *SCNN1B* (Kato et al. 2011). Future research should further investigate variants associated with hypertension risk in Black populations, as these groups are chronically underrepresented in the literature, as well as investigating potential gene-environment interactions influencing hypertension. Additional variants associated with both hypertension and long TL may be revealed in a larger study.

Hypertension is preventable and treatable, through modification of environmental factors like improving diet and increasing physical activity, or antihypertensive medications such as ACE inhibitors or calcium channel blockers (Carey et al. 2022). Hypertension, its complications and associations with CVD cost the NHS approximately £2 billion each year (https://www.gov.uk/government/publications/health-matters-combating-high-bloodpressure/health-matters-combating-high-blood-pressure). Identifying groups most at risk of hypertension as well as individuals with greatest social and environmental risk factors, can help to target interventions and education towards these groups, reducing the burden on the NHS as well as reducing risk factors for CVD in line with the NHS Long Term Plan (https://www.longtermplan.nhs.uk/publication/nhslong-term-plan/). Whilst we focus on BMI and income as readily quantifiable proxies for environmental burden, other factors also contribute to hypertension risk (e.g. psychosocial stress, access to healthcare, or specific metabolic profiles). Nonetheless, BMI and income likely capture broader constructs of social determinants of health. Income may reflect not only healthcare access but also neighbourhood safety, food security, and chronic stress exposure, all of which have been independently linked to cardiovascular risk (Nayak et al. 2020). Our findings should therefore be interpreted as demonstrating that modifiable environmental factors can substantially influence disease outcomes even in the presence of protective genetic factors, rather than as definitive identification of specific causal pathways.

In conclusion, we have shown in the UKB cohort that longer telomeres are indeed protective against many types of CVD in the Black population. However, increased hypertension in Black individuals is seen, where longer telomeres are not protective. We have identified 12 genetic loci in Black individuals that significantly associate with longer telomeres, and key variants in *SLX4* may predispose to both long telomeres and hypertension. Further, social and environmental factors influencing hypertension risk override the effect of the long telomeres in Blacks. In future studies, these ethnicity-specific variants associated with TL should be further investigated, as well as investigating genetic links to hypertension in different ethnic groups.

## Materials and methods

### Study design and participants

Participants in the UKB cohort with leucocyte TL measurements, reported as adjusted T/S ratio (data field (df) 22191_i0), were included. Details of TL measurement are described by Codd et al*.* (2022). who received DNA samples from UK Biobank participants and used qPCR to estimate TL. Based on self-reported ethnic background data (df 21,000) provided at the initial assessment centre, participants were included if ethnicity was reported as White, British, Irish, any other White background (grouped as ‘White’), or Black British, African, Caribbean, any other Black background (grouped as ‘Black’, Figure S1).

To investigate CVD prevalence between ethnicities, summary data on primary and secondary hospital inpatient records (df 41,270) was used to identify individuals with a CVD diagnosis according to the International Classification of Disease version 10 (ICD-10). CVDs included were ischemic heart disease (I20-I25); stroke (I60, I61, I63, I64); atrial fibrillation and flutter (I48); heart failure (I50); and essential (primary) hypertension (I10). Combined CVD and no CVD was also assessed according to diagnoses of chronic rheumatic heart diseases (I05-I09); hypertensive diseases (I10-I15); pulmonary heart disease and diseases of pulmonary circulation (I26-I28); other forms of heart disease (I30-I52); cerebrovascular diseases (I60-I69); and diseases of arteries, arterioles and capillaries (I70-I79).

### TL comparison between ethnicities

To compare TL between ethnicities, mean adjusted T/S ratios were calculated for each ethnicity, and statistical significance was determined with unpaired two-sample T-tests between ethnicities. *P*-values were compared to a threshold value of 0.05 to determine significance, where *p* ≤ 0.05 is statistically significant. To assess the relationship between ethnicity, TL and CVD, mean adjusted T/S ratios for each CVD were compared to no CVD. Statistical significance was determined with unpaired two-sample T tests, where *p* ≤ 0.05 is statistically significant.

### GWAS analysis

To investigate genetic determinants of TL, GWAS was carried out with PLINK2 using the WES data ‘Population level exome OQFE variants, PLINK format final release’, run using Swiss Army Knife on the UKB Research Analysis Platform. In each analysis, participants were excluded if their sex (df 31) and genetic sex (df 22,001) differed; they had sex chromosome aneuploidy (df 22,019), they had genetic kinship to other participants (df 22,021), their samples were not used to compute genetic principal components (df 22,020) or they did not have Whole Exome Sequencing (WES) data available. The WES data was filtered on PLINK2 to remove variants with a minor allele frequency of less than 0.0005; variants with a minor allele count of less than 20; individuals with a genotyping rate of lower than 0.1; variants with a missingness rate of more than 0.1; and variants with a minor allele frequency greater than 0.9995. Linear regression was run on PLINK2 where the phenotype adjusted T/S ratio was used, age (df 21,022) and sex were used as covariates. For the White and dual-ethnicity GWAS, the first ten genetic principal components (df 22,009) were also included as covariates, and the covariates were standardized to mean 0, variance 1. Results and QQ plots were visualised using LocusZoom on the UKB Research Analysis Platform. We excluded results for SNPs with MAF < 0.1, missingness > 5% and HWE *p* < 1 × 10–10).

### Analysis of environmental risk factors associated with CVD and TL

Occurrence of social and environmental factors associated with CVD, particularly hypertension, were investigated between ethnicities. For each factor, individuals who did not answer a question, answered ‘do not know’ or ‘prefer not to answer’ were excluded from analysis. Socioeconomic factors including income were assessed using average total household income before tax | Instance 0 (df 738_i0), measured in GBP annually, education assessed by qualifications | Instance 0 (df 6138_i0), and employment from current employment status | Instance 0 (df 6142_i0). Body mass index (BMI) | Instance 0 (df 21001_i0) data was analysed using The World Health Organisation’s (WHO) criteria to classify BMI into underweight (< 18.5 kg/m^2^), normal weight (18.5 to 24.9 kg/m^2^), overweight (25–29.9 kg/m^2^), and obese (≥ 30.0 kg/m^2^) (Grozdanov et al. 2009). To investigate diet, processed meat intake | Instance 0 (df 1349_i0) and oily fish intake | Instance 0 (df 1329_i0) were used. In each case, participants provide an answer of number of instances eaten per week, considering average intake over the past year. Incidence of T2D was assessed using summary diagnoses (df 41,270) reports for non-insulin-dependent diabetes mellitus (ICD-10 code E11).

## Supplementary Information

Below is the link to the electronic supplementary material.

Supplementary Material 1

## Data Availability

This research was conducted using data from the UK Biobank under approved project number 47602. The UK Biobank dataset is available to researchers upon application (https://www.ukbiobank.ac.uk/enable-your-research/apply-for-access).
