# Supplementary material for: GWAS reveals common SLX4 variants associated with telomere length and hypertension in individuals of African ancestry
Source: Genes Genomics. 2026 Feb 20;48(5):673–82. doi: 10.1007/s13258-026-01746-y (PMC13149702; doi:10.1007/s13258-026-01746-y)
Supplement: Supplementary file 1 — Supplementary Material 1 [file 13258_2026_1746_MOESM1_ESM.pdf]

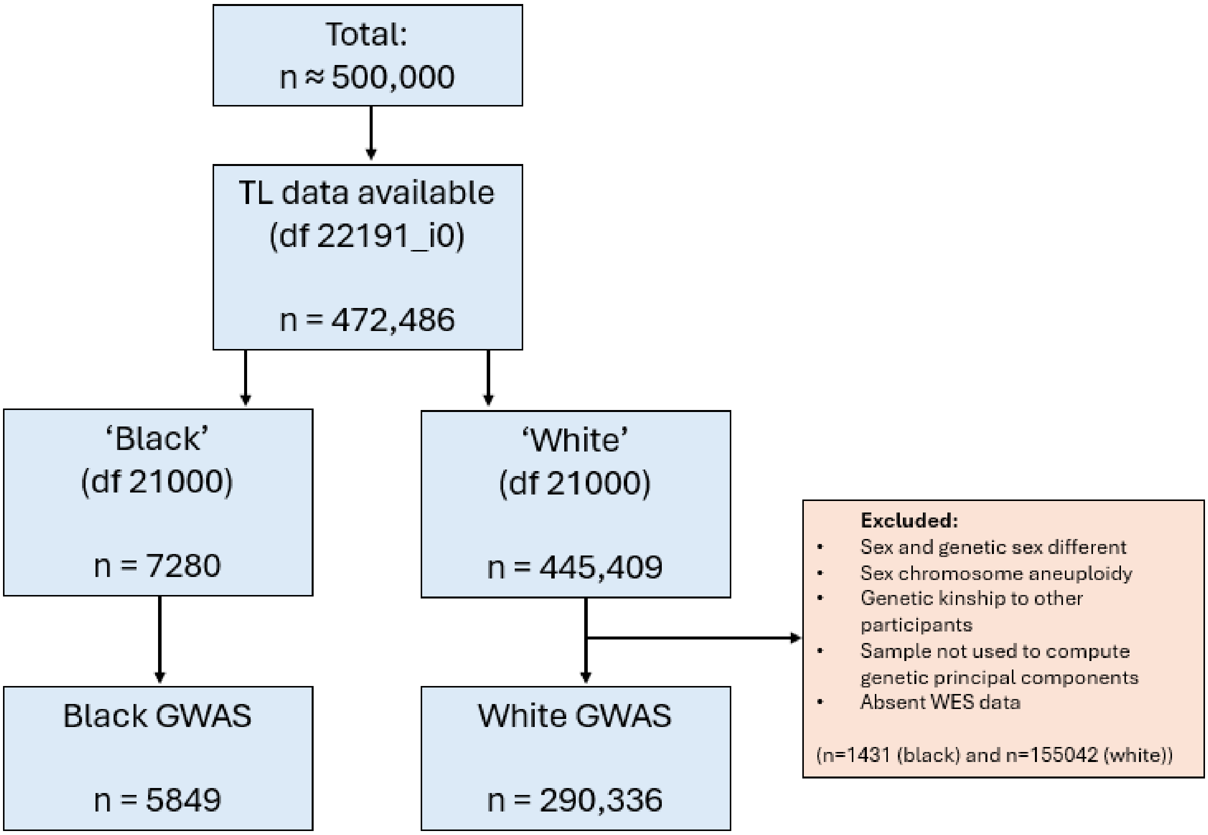

Figure S1 – Filtering of UK Biobank participants for GWAS analysis. Total UK Biobank participants were filtered to measure mean telomere length (TL) and for GWAS analysis. The combined ethnicity GWAS was conducted using Black and White groups (n=296,185). Df = data field.

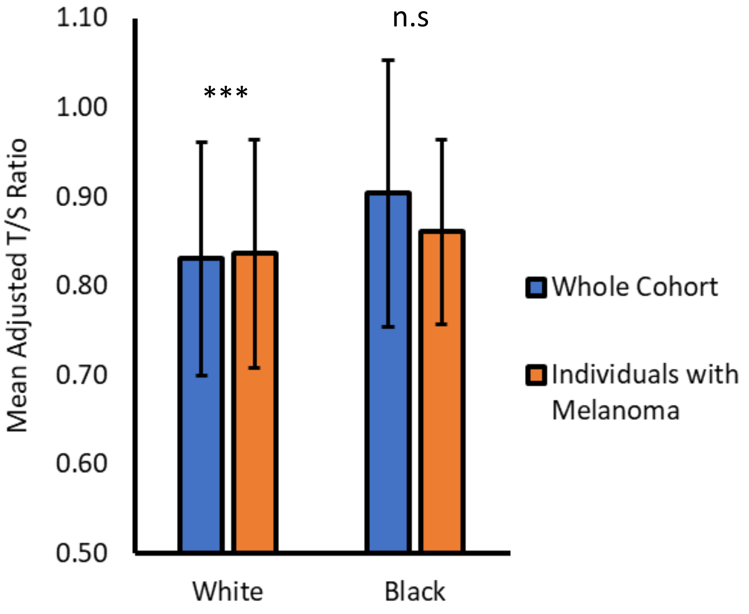

Figure S2 – White individuals with a melanoma diagnosis have significantly longer telomeres than those without melanoma. Mean adjusted T/S ratio in the Black (n=7280) and White (n=445408) cohorts vs individuals presenting with melanoma (White n=4211; Black n=4). Error bars are SEM. *** = p<0.001, unpaired t-test; n.s, not significant.

S.3A

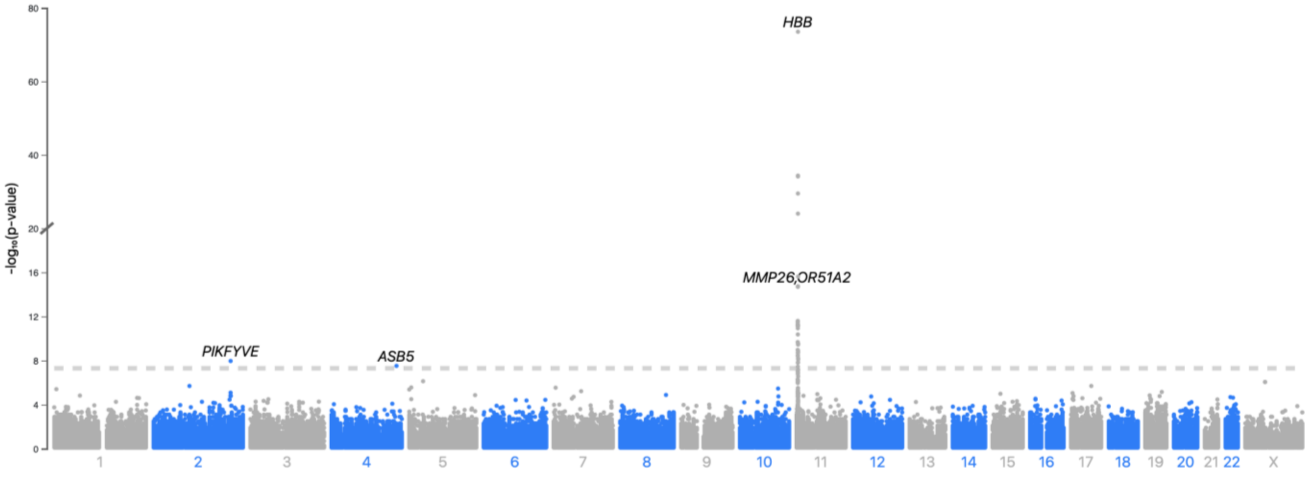

S.3B

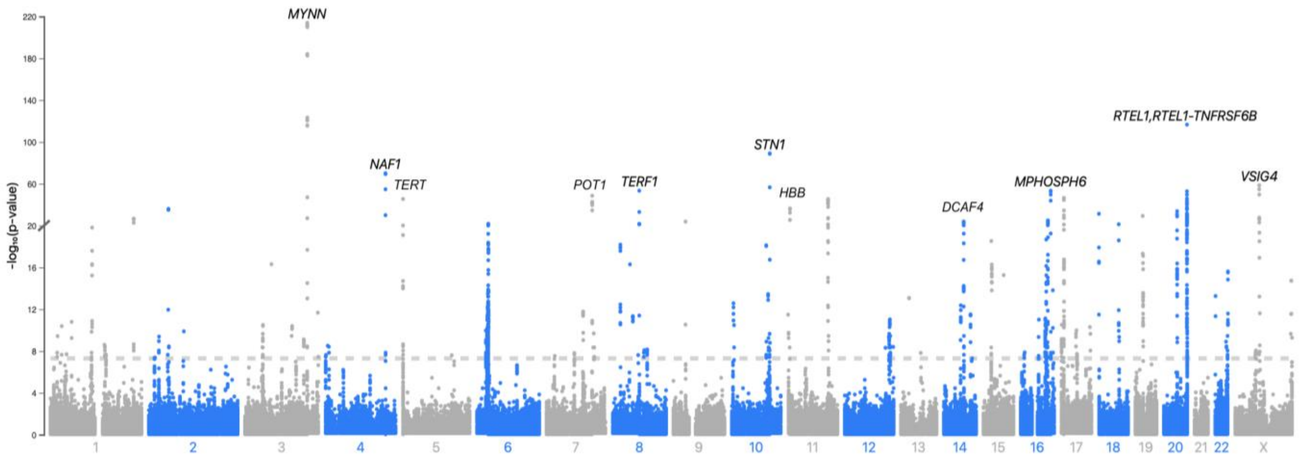

S.3C

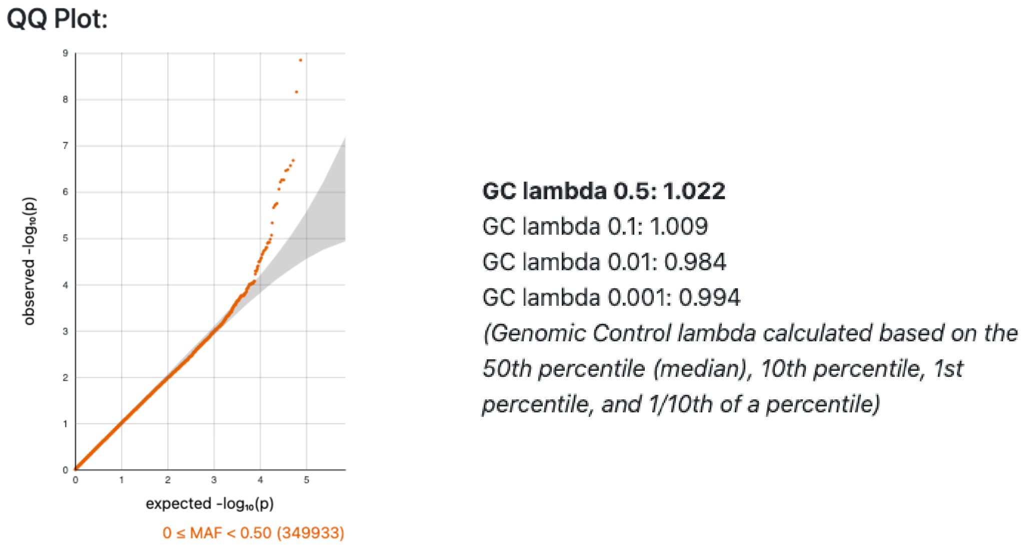

Figure S3 – GWAS analysis identified 106 sentinel variants associated with longer TL in Whites. (S2A) Manhattan plot of significant variants associated with longer telomeres in the Black population following GWAS analysis using WES data (n=5849). (S2B) Manhattan plot of significant variants associated with longer telomeres in the white population following GWAS analysis using WES data (n=290,366). Horizontal dashed reference line = GWAS significance threshold. WES; whole exome sequencing. (S2C) QQ plot for multi-ethnicity GWAS presented in Figure 2.

S4.A

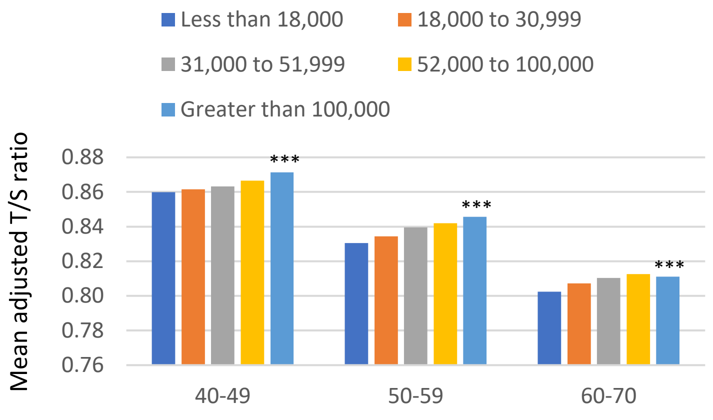

Age Category	Income	Count	Mean T/S	StDev T/S
40-49	Less than 18,000	10626	0.8598	0.1300
	18,000 to 30,999	16241	0.8616	0.1341
	31,000 to 51,999	28384	0.8632	0.1320
	52,000 to 100,000	27945	0.8665	0.1377
	Greater than 100,000	7934	0.8714	0.1351
50-59	Less than 18,000	20090	0.8305	0.1284
	18,000 to 30,999	28437	0.8344	0.1268
	31,000 to 51,999	38500	0.8396	0.1284
	52,000 to 100,000	34616	0.8419	0.1297
	Greater than 100,000	9152	0.8456	0.1272
60-70	Less than 18,000	54675	0.8025	0.1252
	18,000 to 30,999	52890	0.8071	0.1254
	31,000 to 51,999	33910	0.8103	0.1248
	52,000 to 100,000	16293	0.8126	0.1250
	Greater than 100,000	3846	0.8111	0.1239

S4.B

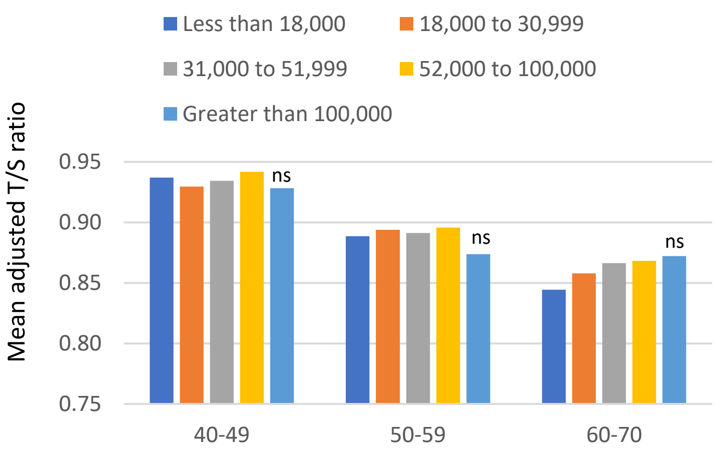

Age Category	Income	Count	Mean T/S	StDev T/S
40-49	Less than 18,000	807	0.9369	0.1508
	18,000 to 30,999	732	0.9296	0.1485
	31,000 to 51,999	677	0.9344	0.1527
	52,000 to 100,000	384	0.9418	0.1703
	Greater than 100,000	47	0.9283	0.1179
50-59	Less than 18,000	624	0.8886	0.1383
	18,000 to 30,999	625	0.8940	0.1402
	31,000 to 51,999	458	0.8911	0.1371
	52,000 to 100,000	232	0.8958	0.1397
	Greater than 100,000	31	0.8736	0.1491
60-70	Less than 18,000	522	0.8443	0.1326
	18,000 to 30,999	253	0.8580	0.1316
	31,000 to 51,999	135	0.8664	0.1475
	52,000 to 100,000	46	0.8682	0.1433
	Greater than 100,000	8	0.8721	0.1245

Supplementary figure 4 – Telomere length increases with income when stratified by age. (A) Mean adjusted telomere length for Whites per income bracket, stratified by age. (B) Mean adjusted telomere length for Blacks per income bracket, stratified by age. *** = P<0.0001 (unpaired t-test) when comparing lowest to highest income in the group. ns = not significant.

S5.A

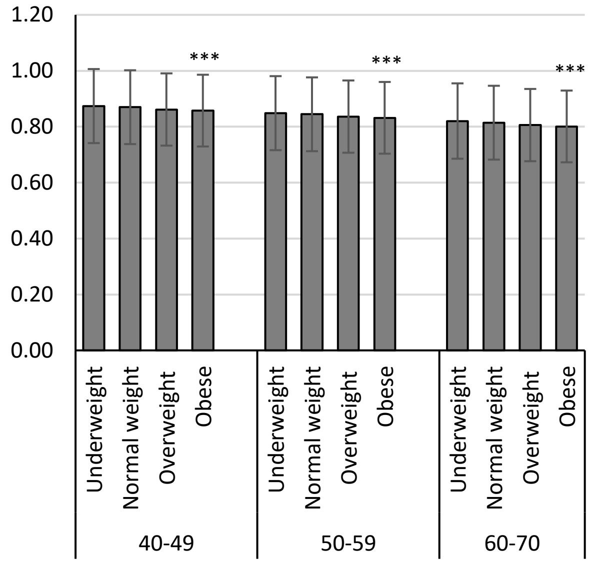

Age Category	Income	Count	Mean T/S	StDev T/S
40-49	Underweight	3126	0.8737	0.1324
	Normal weight	35568	0.8698	0.1320
	Overweight	39194	0.8615	0.1292
	Obese	21728	0.8575	0.1283
50-59	Underweight	3528	0.8484	0.1324
	Normal weight	46152	0.8444	0.1320
	Overweight	60519	0.8361	0.1292
	Obese	37495	0.8317	0.1283
60-70	Underweight	3607	0.8202	0.1348
	Normal weight	55336	0.8143	0.1320
	Overweight	89325	0.8058	0.1292
	Obese	49830	0.8009	0.1283

S5.B

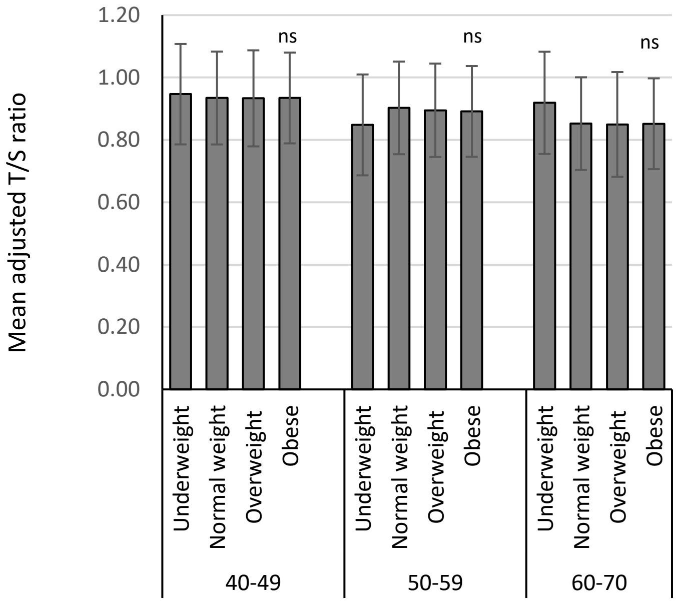

Age Category	Income	Count	Mean T/S	StDev T/S
40-49	Underweight	37	0.9465	0.1609
	Normal weight	656	0.9342	0.1488
	Overweight	1363	0.9332	0.1540
	Obese	1276	0.9343	0.1455
50-59	Underweight	20	0.8480	0.1617
	Normal weight	392	0.9025	0.1485
	Overweight	982	0.8948	0.1498
	Obese	1048	0.8911	0.1456
60-70	Underweight	8	0.9187	0.1639
	Normal weight	242	0.8521	0.1486
	Overweight	606	0.8494	0.1680
	Obese	650	0.8517	0.1457

Supplementary figure 4 – Telomere length decreases with increased BMI when stratified by age. (A) Mean adjusted telomere length for Whites per BMI group, stratified by age. (B) Mean adjusted telomere length for Blacks per BMI group, stratified by age. *** = P<0.0001 (unpaired t-test) when comparing to underweight BMI. ns = not significant.

Table S1: Sentinel variants associated with longer TL identified in White GWAS analysis

Marker	rsID	Nearest Gene	-log(p)	p	Loci	Variant
3: 169,774,313 C/T	rs10936599	*MYNN*	213.925	1.19E-214	Known	Known
20: 63,689,775 G/A	rs35640778	*RTEL1, RTEL1-TNFRSF6B*	116.619	2.40E-117	Known	Known
10: 103,897,558 G/C	rs10786775	*STN1*	89.088	8.17E-90	Known	Known
4: 163,127,047 G/C	rs4691896	*NAF1*	70.141	7.23E-71	Known	Novel
X: 66,027,567 T/A	rs9633155	*VSIG4*	58.555	2.79E-59	Known	Novel
8: 73,046,129 G/A	rs145702999	*TERF1*	53.422	3.78E-54	Known	Novel
16: 82,170,106 G/T	rs2303264	*MPHOSPH6*	53.395	4.03E-54	Known	Novel
7: 124,835,242 T/TAAACA	rs66826272	*POT1*	48.576	2.65E-49	Known	Novel
17: 7,857,079 G/A	rs4724	*NAA38*	46.33	4.68E-47	Novel	Novel
5: 1,293,971 C/T	rs2736098	*TERT*	45.476	3.34E-46	Known	Known
11: 108,474,788 G/GA	rs11374964	*POGLUT3*	45.385	4.12E-46	Novel	Novel
11: 5,226,561 C/G	rs10768683	*HBB*	36.409	3.90E-37	Known	Novel
2: 54,255,416 G/A	rs17189743	*ACYP2, TSPYL6*	35.925	1.19E-36	Known	Known
20: 37,032,643 A/T	rs6017076	*RBL1*	33.765	1.72E-34	Novel	Novel
18: 672,836 A/G	rs1059393	*TYMS, ENOSF1*	31.317	4.82E-32	Known	Novel
19: 21,109,642 C/A	rs7248410	*ZNF714*	29.319	4.80E-30	Novel	Novel
1: 226,407,946 C/G	rs907187	*PARP1*	26.555	2.79E-27	Known	Known
16: 74,675,308 G/A	rs11862413	*MLKL*	24.845	1.43E-25	Novel	Novel
14: 72,938,044 G/C	rs2302588	*DCAF4*	23.868	1.36E-24	Known	Known
9: 34,107,507 C/T	rs11557154	*DCAF12*	23.852	1.41E-24	Novel	Novel
6: 31,815,730 G/C	rs1043618	*HSPA1A*	21.772	1.69E-22	Known	Novel
18: 54,269,477 G/T	rs3730668	*AC093462.1, POLI*	21.3	5.01E-22	Known	Known
1: 113,901,277 G/A	rs17464525	*AP4B1*	19.812	1.54E-20	Novel	Novel
X: 66,605,144 C/T	rs1385699	*EDA2R*	19.327	4.71E-20	Novel	Novel
16: 70,138,870 G/A	rs114934918	*PDPR, AC009060.1*	18.677	2.10E-19	Novel	Novel
15: 40,611,486 A/G	rs12911738	*KNL1*	18.529	2.96E-19	Novel	Novel
8: 21,989,075 G/C	rs2306645	*XPO7*	18.179	6.62E-19	Known	Novel
10: 94,355,078 G/A	rs12572897	*NOC3L*	18.118	7.62E-19	Novel	Novel
19: 22,193,066 C/T	rs8104929	*ZNF676*	17.114	7.69E-18	Known	Novel
17: 8,236,442 T/C	rs58532865	*CTC1*	16.76	1.74E-17	Known	Novel
3: 72,842,396 A/C	rs78491606	*SHQ1*	16.313	4.86E-17	Known	Known
8: 47,972,876 T/A	rs762679	*MCM4*	16.295	5.07E-17	Known	Known
15: 41,740,185 C/G	rs17677991	*MGA*	16.275	5.31E-17	Novel	Novel
22: 50,522,740 A/G	rs131813	*NCAPH2*	15.608	2.47E-16	Known	Novel
15: 74,044,292 T/C	rs5742915	*PML*	15.268	5.40E-16	Known	Known
X: 152,767,196 C/A	rs61744011	*MAGEA6*	14.74	1.82E-15	Known	Novel
16: 87,975,106 C/CT	rs71156278	*BANP*	13.815	1.53E-14	Known	Novel
10: 99,533,278 C/A	rs41290504	*NKX2-3*	13.449	3.56E-14	Known	Novel
22: 16,988,159 C/A	rs28502153	*GAB4*	13.267	5.41E-14	Known	Novel
13: 41,193,202 A/G	rs2039134	*AL354696.2, KBTBD7*	13.087	8.18E-14	Known	Novel
10: 5,785,111 G/A	rs10795547	*GDI2*	12.572	2.68E-13	Novel	Novel
14: 65,076,384 C/T	rs45604339	*MAX*	12.371	4.26E-13	Known	Novel
6: 30,103,553 C/T	rs1116221	*TRIM31*	12.211	6.15E-13	Novel	Novel
2: 53,765,485 G/C	rs36020289	*ASB3*	11.958	1.10E-12	Novel	Novel
7: 100,180,604 G/T	rs11531577	*STAG3*	11.768	1.71E-12	Known	Novel
3: 198,153,259 GGCAGCAGCA/G	-	*FAM157A*	11.676	2.11E-12	Novel	Novel
X: 67,695,821 T/A	rs188619593	*AR*	11.613	2.44E-12	Novel	Novel
14: 91,462,648 T/G	rs5020186	*PPP4R3A*	11.511	3.08E-12	Known	Novel
11: 197,337 G/A	rs3802984	*BET1L, ODF3*	11.483	3.29E-12	Novel	Novel
8: 55,796,142 T/C	rs138207138	*TGS1*	11.337	4.60E-12	Known	Novel
16: 67,658,960 T/C	rs139438549	*ACD*	11.118	7.62E-12	Known	Known
12: 123,326,326 G/A	rs61388686	*SBNO1*	11.029	9.35E-12	Novel	Novel
16: 50,231,042 C/CTTTA	rs3887556	*TENT4B*	11.01	9.77E-12	Known	Novel
6: 32,832,444 C/T	rs2856992	*AL669918.1, TAP2*	10.806	1.56E-11	Novel	Novel
1: 58,539,307 C/T	rs902925	*DAB1, OMA1*	10.797	1.60E-11	Novel	Novel
17: 1,879,658 A/G	rs5030755	*RPA1*	10.52	3.02E-11	Known	Known

Table S1 continued

3: 49,898,669 T/C	rs2230590	*MST1R*	10.497	3.18E-11	Novel	Novel
10: 7,292,138 G/A	rs749165	*SFMBT2*	10.484	3.28E-11	Novel	Novel
16: 68,249,467 AG/A	rs545651676	*PLA2G15*	10.418	3.82E-11	Novel	Novel
3: 128,638,009 C/T	rs1697	*RPN1*	10.399	3.99E-11	Known	Novel
1: 31,814,028 G/A	rs6669563	*SPOCD1*	10.386	4.11E-11	Known	Known
12: 122,602,895 A/G	rs7307735	*KNTC1*	10.295	5.07E-11	Novel	Novel
17: 78,190,894 C/T	rs28712867	*AFMID*	10.292	5.11E-11	Novel	Novel
6: 25,420,116 C/G	rs913455	*CMAHP, CARMIL1*	9.983	1.04E-10	Known	Known
17: 41,827,556 G/C	rs1046404	*NT5C3B*	9.976	1.06E-10	Novel	Novel
6: 26,021,644 C/T	rs41266779	*HIST1H4A*	9.953	1.11E-10	Novel	Novel
6: 28,360,614 CTT/C	rs34491374	*ZKSCAN3*	9.929	1.18E-10	Novel	Novel
2: 95,950,832 A/G	rs75927533	*ANKRD36C*	9.89	1.29E-10	Novel	Novel
16: 67,189,100 G/A	rs138207138	*EXOC3L1*	9.89	1.29E-10	Novel	Novel
7: 128,947,297 CACTCTGCAGCCGCCCACTCTGCGGCCGCCT/C	rs60344245	*IRF5*	9.691	2.04E-10	Known	Novel
16: 69,330,540 A/G	rs8057004	*COG8, PDF, AC026464.6*	9.657	2.20E-10	Novel	Novel
1: 20,589,096 TC/T	rs3215400	*CDA*	9.431	3.71E-10	Known	Novel
2: 28,864,933 C/A	rs13030945	*TRMT61B*	9.384	4.13E-10	Known	Novel
12: 120,469,468 G/T	rs11548262	*SRSF9*	9.352	4.45E-10	Known	Novel
X: 154,791,839 C/A	rs1126762	*MPP1*	9.277	5.28E-10	Novel	Novel
3: 160,417,640 C/T	rs1451760	*AC079594.2, SMC4*	9.117	7.64E-10	Known	Novel
10: 94,762,608 T/C	rs4986894	*AL583836.1*	8.903	1.25E-09	Novel	Novel
22: 45,387,023 T/C	rs6007010	*SMC1B*	8.901	1.26E-09	Known	Novel
10: 104,403,775 C/T	rs41291850	*CFAP58*	8.791	1.62E-09	Novel	Novel
1: 147,242,777 G/C	rs11588753	*AC242426.2, FMO5, CHD1L*	8.604	2.49E-09	Known	Novel
4: 7,042,653 C/T	rs871134	*AC097382.2, CCDC96, TADA2B*	8.508	3.10E-09	Known	Known
4: 9,996,816 C/T	rs10939650	*SLC2A9*	8.417	3.83E-09	Known	Novel
12: 132,490,757 A/G	rs4883578	*FBRSL1*	8.4	3.98E-09	Novel	Novel
3: 48,187,876 C/A	rs146179438	*CDC25A*	8.372	4.25E-09	Novel	Novel
12: 111,446,804 T/C	rs3184504	*SH2B3, ATXN2*	8.316	4.83E-09	Novel	Novel
10: 102,424,324 G/A	rs1044476	*CUEDC2*	8.244	5.70E-09	Novel	Novel
8: 94,534,891 T/G	rs2304764	*VIRMA*	8.131	7.40E-09	Known	Novel
8: 86,556,416 T/G	rs2304787	*CPNE3, CNGB3*	8.082	8.28E-09	Novel	Novel
X: 57,292,474 T/A	rs4030473	*FAAH2*	8.057	8.77E-09	Novel	Novel
X: 56,268,812 T/C	rs3747284	*KLF8*	7.922	1.20E-08	Novel	Novel
16: 66,912,352 C/T	rs147611353	*CDH16*	7.904	1.25E-08	Novel	Novel
16: 11,915,447 C/A	rs11544193	*GSPT1*	7.875	1.33E-08	Novel	Novel
13: 72,766,039 G/A	rs35017269	*DIS3*	7.825	1.50E-08	Known	Known
7: 76,515,166 A/G	rs1636632	*UPK3B*	7.815	1.53E-08	Known	Novel
17: 43,178,007 T/C	rs11657883	*NBR1*	7.729	1.87E-08	Novel	Novel
1: 44,784,335 C/G	rs59895800	*BEST4*	7.701	1.99E-08	Known	Novel
X: 65,534,764 A/G	rs188902388	*LAS1L*	7.668	2.15E-08	Novel	Novel
1: 151,401,549 T/C	rs4603	*PSMB4*	7.664	2.17E-08	Known	Novel
4: 2,251,253 T/G	rs2304764	*MXD4*	7.634	2.32E-08	Novel	Novel
8: 70,068,503 T/C	rs3750228	*PRDM14*	7.616	2.42E-08	Known	Novel
5: 133,091,159 GTT/G	rs147133967	*HSPA4*	7.601	2.51E-08	Known	Novel
7: 23,710,213 G/T	rs3807888	*STK31*	7.534	2.92E-08	Known	Novel
6: 27,807,896 A/G	rs200484	*HIST1H2BL*	7.475	3.35E-08	Novel	Novel
10: 103,326,511 TC/T	rs560179122	*PCGF6*	7.416	3.84E-08	Novel	Novel
3: 48,564,341 A/G	rs74780677	*COL7A1*	7.402	3.96E-08	Novel	Novel
2: 17,781,726 CAAGTT/C	rs149936944	*SMC6, GEN1*	7.381	4.16E-08	Known	Novel
